# Tracing the dynamics of superconducting order via transient terahertz third-harmonic generation

**DOI:** 10.1126/sciadv.adi7598

**Published:** 2024-03-15

**Authors:** Min-Jae Kim, Sergey Kovalev, Mattia Udina, Rafael Haenel, Gideok Kim, Matteo Puviani, Georg Cristiani, Igor Ilyakov, Thales V. A. G. de Oliveira, Alexey Ponomaryov, Jan-Christoph Deinert, Gennady Logvenov, Bernhard Keimer, Dirk Manske, Lara Benfatto, Stefan Kaiser

**Affiliations:** ^1^ Institute of Solid State and Materials Physics, TUD Dresden University of Technology, 01069 Dresden, Germany.; ^2^ Max Planck Institute for Solid State Research, 70569 Stuttgart, Germany.; ^3^ 4th Physics Institute and Research Center SCoPE, University of Stuttgart, 70569 Stuttgart, Germany.; ^4^ Helmholtz-Zentrum Dresden-Rossendorf, 01328 Dresden, Germany.; ^5^ Department of Physics and ISC-CNR, “Sapienza” University of Rome, 00185 Rome, Italy.; ^6^ Department of Physics and Astronomy & Stewart Blusson Quantum Matter Institute, University of British Columbia, Vancouver, BC V6T 1Z4, Canada.

## Abstract

Ultrafast optical control of quantum systems is an emerging field of physics. In particular, the possibility of light-driven superconductivity has attracted much of attention. To identify nonequilibrium superconductivity, it is necessary to measure fingerprints of superconductivity on ultrafast timescales. Recently, nonlinear THz third-harmonic generation (THG) was shown to directly probe the collective degrees of freedoms of the superconducting condensate, including the Higgs mode. Here, we extend this idea to light-driven nonequilibrium states in superconducting La_2-*x*_Sr*_x_*CuO_4_, establishing an optical pump–THz–THG drive protocol to access the transient superconducting order-parameter quench and recovering on few-picosecond timescales. We show in particular the ability of two-dimensional TH spectroscopy to disentangle the effects of optically excited quasiparticles from the pure order-parameter dynamics, which are unavoidably mixed in the pump-driven linear THz response. Benchmarking the gap dynamics to existing experiments shows the ability of driven THG spectroscopy to overcome these limitations in ordinary pump-probe protocols.

## Teaser

High-field THz pulses measure nonlinear excitations that directly probe the ultrafast dynamics of light-driven superconductors.

## INTRODUCTION

The ability of short light pulses to stabilize quasi-ordered states not accessible in thermal equilibrium demonstrated its potential in a variety of fields. Resonant ultrafast excitation of atomic oscillations controls the electronic properties of materials, leading to remarkable phenomena such as switching of ferroelectricity and multiferroicity (*1*–*4*) and light-induced superconductivity (*5*–*8*). In the case of ordered states formed via the spontaneous breaking of a preexisting symmetry, time-resolved protocols turned out to be crucial to address the physical mechanisms at play in unconventional systems, which deviate from the standard mean field–like description. A paradigmatic case is provided by high-temperature superconductors like cuprates, where superconductivity emerges out of a complex and strongly correlated normal state with marked signatures of fluctuating charge and spin order (*9*). In this situation, standard spectroscopic probes can fail in providing a clear-cut signature of the pure superconducting (SC) order and of its dynamical evolution. At the same time, time-resolved protocols in both conventional and unconventional superconductors revealed so far rather different dynamics depending on the wavelength of the initial pump pulse. The ability of a short light pulse to quench the SC order is partly hindered by the simultaneous creation of quasiparticle excitations, leading to a mixing among the two signals that can be hard to disentangle (*10*–*14*).

Here, we outline a specific approach to pinpoint the dynamics of the SC order parameter via transient detection of THz-driven third-harmonic (TH) generation (THG). In standard THG measurements, a high-field multicycle THz pulse of frequency ω below the SC gap enforces driven oscillations of the SC condensate with twice the driving frequency, leading to a characteristic THG signal at frequency 3ω. As shown by previous work, the TH intensity can be well explained by a quasi-equilibrium approach, where the strength of the 3ω response scales with a nonlinear optical kernel χ, which grows below *T*_c_ as the equilibrium order parameter, χ ∼ Δ, of the condensate. In the SC state, χ is dominated by Cooper-pair and Higgs mode excitations at 2∆, i.e., twice the equilibrium value of the SC order parameter (*15*–*24*). In the present work, we use a second intense optical pump to modulate the SC ground state and then dynamically access the transient evolution of the THG. We show that by means of the simultaneous control over the gate acquisition time and the pump-probe delay, we can efficiently disentangle the quasiparticle pair-breaking effects from the pure time evolution of ∆(*t*). Further, realizing a well-understood quench protocol of the condensate, we probe its recovery on a picosecond timescale. Our experiment demonstrates the high potential of two-dimensional (2D) optical-THz pump-drive experiments to disentangle the various degrees of freedom at play in a photoexcited process via a selective identification of the relevant excitation mechanisms.

## RESULTS

**Fig. 1.**
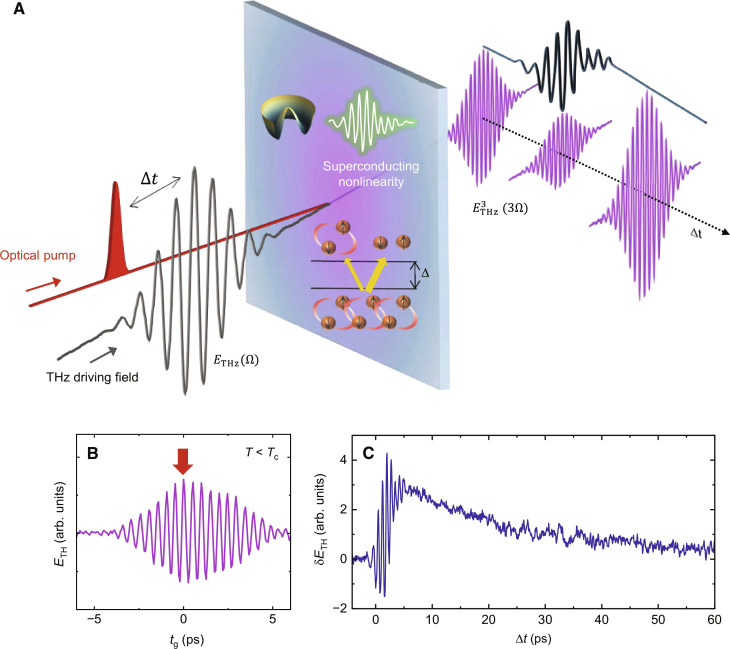
Optical pump–TH drive signal in a superconducting LSCO thin film. (**A**) Experimental scheme: An optical pump pulse triggers the pair breaking dynamics in the superconductor. A time-delayed intense multicycle THz driving field *E*_THz_ (Ω) drives a THG *E*^3^_THz_ (3Ω) that probes the transient nonlinear dynamics at a pump time delay of ∆*t* after the excitation pulse. The transient dynamics of the TH field results in a superposition of intrinsic THG changes of the SC condensate itself and contributions of nonlinear currents due to the pair breaking process and the generation of quasiparticles. The dynamics can be probed as 1D signal of the peak TH field changes δ*E*_TH_ as a function of the pump-drive delay ∆*t* [signal along ∆*t*, (C)] or as 2D signal of the transient THG at each time delay (Figs. 2 and 4). (**B**) Generated TH field without the optical pump pulse as a function of an internal THz gate delay time *t*_g_ at the equilibrium SC state *T < T*_c_. (**C**) 1D signal of the photo-induced TH field changes δ*E*_TH_ as a function of pump-drive time delay ∆*t* measured at the equilibrium peak field position indicated by the red arrow in (B). The onset of the signal shows pronounced oscillations at 2Ω and 4Ω of the driving THz field.

In this study, we report the light-induced nonlinear THz response of photoexcited La_2-*x*_Sr*_x_*CuO_4_, a high-*T*_c_ SC cuprate. We extend the THz-driven THG experiments (*16*, *25*) into a pump-drive experiment in the time domain as sketched in Fig. 1A. As done in previous studies (*25*), we initially generate TH without optical excitation shown as an electro-optical (EO) sampling trace with the internal gate delay time *t*_g_ in Fig. 1B: A high-field multicycle THz drive *E*_THz_ (Ω) at ω = 0.7 THz (2.5 meV) far below the SC gap as driving pulse generates a TH field *E*_THz_ (3Ω) at ω = 2.1 THz in the SC La_2-*x*_Sr*_x_*CuO_4_ thin film at 12 K [*T*_c_ = 44 K, ∆_SC_ ~ 20 meV (*26*)]. As previous experimental (*16*, *17*, *25*, *27*–*31*) and theoretical work (*15*–*24*, *32*, *33*) demonstrated, the thermally induced transition to the SC state strongly enhances the THG both in conventional and unconventional superconductors, making THG a preferential tool to probe the coherent SC state. Within a quasi-equilibrium approach, the THG is controlled by the nonlinear current *j*_NL_ ~ χ*E*^3^, where χ is the nonlinear kernel and *E* is the local electric field within the sample. In the presence of a long-range SC order parameter ∆, the nonlinear response χ shows a marked resonance at 2*∆* due to both particle-hole Bardeen–Cooper–Schrieffer (BCS) excitations and Higgs oscillations on top of the equilibrium SC ground state. Their relative importance depends on various material parameters, like, e.g., disorder (*19*, *20*, *24*) and correlations (*22*), and they can coexist with additional low-energy SC fluctuations as, e.g., Leggett (*29*, *32*) and soft plasma modes (*34*). On general grounds, it is well established that in single-band superconductors, the THG enhancement below *T*_c_ follows the rising of ∆, i.e., χ ∼ Δ, regardless of the excitation mechanism dominating the equilibrium THG response*.* In our experiment, a femtosecond optical pump pulse at 1.55 eV excites the superconductor and the light-induced changes of the TH signal probe the triggered nonequilibrium dynamics of the condensate. The transient dynamics is scanned by varying the time delay ∆*t* between the pump and drive pulses. Two experimental protocols are possible. In a 2D scan, full TH transients are measured for different pump delays. In a 1D scan, only the pump beam is moved with respect to the TH field that is probed at a specific position. For the experimental details, see text S1.

Figure 1C shows the photoexcited TH signal dynamics measured as a 1D scan taken as changes in the peak amplitude of the unperturbed TH field *E*_THz_ (3Ω) (marked by the arrow in Fig. 1B) in the SC state below *T*_c_. Varying the pump-drive delay ∆*t*, the TH sampling position remains fixed. The differential TH signal δ*E*_TH_ is characterized by an increase of the THG, with a long-lasting incoherent excitation dynamics and an additional oscillatory signal at twice (2Ω) and fourth (4Ω) the frequency of the driving field (Ω) at early pump-drive time delay (text S2). In the following, such higher harmonics will be characterized as sidebands at (3Ω ± Ω) of the original TH signal due to a wave mixing with the visible pump pulse. This is done via a 2D spectroscopic approach measuring the generated transient TH at different pump-drive delays ∆*t* (Fig. 1A).

The incoherent dynamics is characterized by a rise time of ~5 ps and an initial decay time of about 20 ps by a biexponential fit for a low pump excitation fluence of 15 μJ/cm^2^. Following the initial decay, the temporal changes in TH signal occur on a notably longer timescale. This long-relaxation response mimics the linear THz and optical response, attributed to the recombination processes between excited incoherent quasiparticles and phonons (*10*, *14*). However, TR-ARPES (time- and angle-resolved photoemission spectroscopy) shows an excitation and relaxation dynamics of the gap with comparatively much faster relaxation times, in the low picosecond range, as shown in Bi_2_Sr_2_CaCu_2_O_8+δ_ (BSCCO) (*35*). The increase of the TH signal is at first sight rather unexpected: THG measurements at equilibrium demonstrated that the nonlinear kernel χ decreases by warming in the SC state, suggesting that partial melting of SC order due to the pump should rather lead to a suppression of THG (*36*). Such an observation, along with the emergence of sideband oscillations of the original TH response, can be understood by modeling the differential TH signal as

$$\delta E_{\text{TH}}\left(\Delta t\right)∼\chi^{\left(3\right)}E_{\text{THz}}E_{\text{pump}}^{2}$$ (1)

where the exact time convolution is detailed in text S3. According to Eq. 1, one needs to carefully examine the effect of the pump not only on the nonlinear kernel χ but also on the local THz field *E*_THz_ that can be nontrivially modified by the incoherent dynamics of pump-induced excited quasiparticles. As a consequence, the extraction of the pure nonlinear response requires a proper normalization of the signal (*25*), as we will discuss below.

**Fig. 2.**
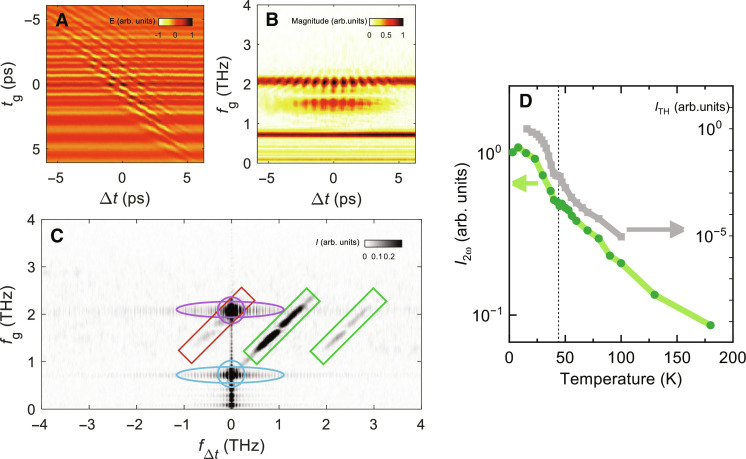
2D signal of the nonlinear dynamics in the photoexcited LSCO. (**A**) Transient dynamics of the transmitted THz field *E* (*t*_g_, ∆*t*) at 5 K after the excitation with an optical pump fluence *F*_pump_ = 20 μJ/cm^2^. The spectrum is normalized by the maximum value of the transient THz field. (**B**) Transient spectrum after a Fourier transformation of (A) along the THz gate time axis *t*_g_. The spectrum is normalized by the equilibrium value of the FH magnitude. (**C**) 2D spectrum after transforming along gate time axis *t*_g_ and pump-drive delay axis ∆*t*. The circles mark the components of the fundamental drive and the THG signal at equilibrium, and the cigar-shaped marks represent the transient incoherent dynamics of the fundamental and TH field due to the optical pump pulse. The rectangular boxes mark a sideband generation that emerges from a modulation of the 3Ω TH signal with an Ω signal of quasiparticles in the driving THz field and higher orders, as seen by the oscillations at early times in the 1D scans of Fig. 1C. The discontinuous feature in the middle of the sideband is due to band-pass filters in the experimental setup (see text S3). (**D**) Intensity of the coherent 2Ω oscillation of the 3Ω TH signal (*I*_2ω_) compared to the static TH intensity *I*_TH_ [data from (*25*)] as a function of temperature. The black dashed line marks the critical temperature *T*_c_.

To characterize the spectral components of the differential TH signal, we perform 2D scans to obtain the full-2D spectrum of the transient TH dynamics as a function of the internal gate time of the EO sampling, *t*_g_, and the pump-drive delay time ∆*t*. Figure 2A shows this spectrum in the time domain for field strength of 100 kV/cm of the multicycle THz pulse and a pump fluence of *F*_pump_ = 20 μJ/cm^2^ of the femtosecond optical pump. The vertical pattern along *t*_g_ for a fixed ∆*t* shows the response to the THz drive: As in the nonpumped experiments (*25*), the transmitted THz signal shows oscillations as a function of *t*_g_ consisting of a superposition of the fundamental harmonic (FH) and the TH component generated by the nonlinear response inside the superconductor. They are both clearly visible in Fig. 2B, where the vertical axis *f*_g_ is the Fourier transform of the gate time, and marked bands appear at *f*_FH_ = 0.7 THz and *f*_TH_ = 2.1 THz, respectively. When the optical pump sets in, a modulation of the TH along ∆*t* appears, persisting for the pulse duration of the drive. The modulation frequency of the TH signal, already seen at early times in the 1D scans in Fig. 1C, has a strong component at 2Ω and a smaller one at 4Ω of the driving pulse (see text S2). In addition, a strong transient signal at a frequency lower than 3Ω around *f*_g_ = 1.4 THz emerges in Fig. 2B. Figure 2C shows the response along the 2D Fourier-transformed coordinates, *f*_g_ and *f*_∆*t*_, respectively. Here, we can disentangle the different contributions arising from THz high-harmonic generation, transient incoherent dynamics, and THz-optical wave mixing processes, naturally encoded in Eq. 1. The FH and TH of the driving THz field at *f_∆t_* = 0 are marked by the blue and violet circles. These are the same features present in the nonpumped experiment (*25*). The pump-probe incoherent dynamics responsible for the long-lasting increase of δ*E*_TH_ shown in Fig. 1C contributes to the regions highlighted by the blue and violet cigar shapes. The previously unknown harmonical features that we observe, both the TH modulation in Figs. 1C and 2B, as well as the previously unknown spectral feature around 1.4 THz in Fig. 2B, can be understood as sideband processes in the periodically driven system. Such a response, absent in the transient optical pump THz probe experiment (*14*), can be understood by considering four- and six-wave mixing processes among the THz and optical pulses (see text S3), leading to spectral features at *f*_g_ = *f*_∆*t*_ ± Ω (green rectangles) and *f*_g_ = *f_∆t_* ± 3Ω (red rectangle). The four-wave mixing process, which scales as Eq. 1, is responsible for a modulation of the TH signal with a periodicity of 2Ω (and 4Ω at higher order), while the drive pulse is present. The spectral width of the sidebands is mainly set by the width of the difference-frequency process around zero frequency of the femtosecond optical pulse.

Notice that the present second harmonic (SH) modulation of the TH signal, *I*_2ω_, can still be fully captured within a quasi-equilibrium approach, as shown in Fig. 2D, and it is not linked to any (static of dynamically generated) additional zero-frequency field component (Fig. 2C), usually invoked to explain SH generation of the original driving field in nonpumped experiments (*30*, *37*, *38*). In Fig. 2D, we show its temperature dependence (green, in log scale), alongside with the static TH field intensity [gray, linear scale, from (*2*5)]. Both decrease as the temperature increases, and they are both detected even above *T*_c_. The nonvanishing TH signal above *T*_c_ has been previously reported (*25*) and may be linked to SC fluctuations of various origins (*34*, *39*–*41*).

**Fig. 3.**
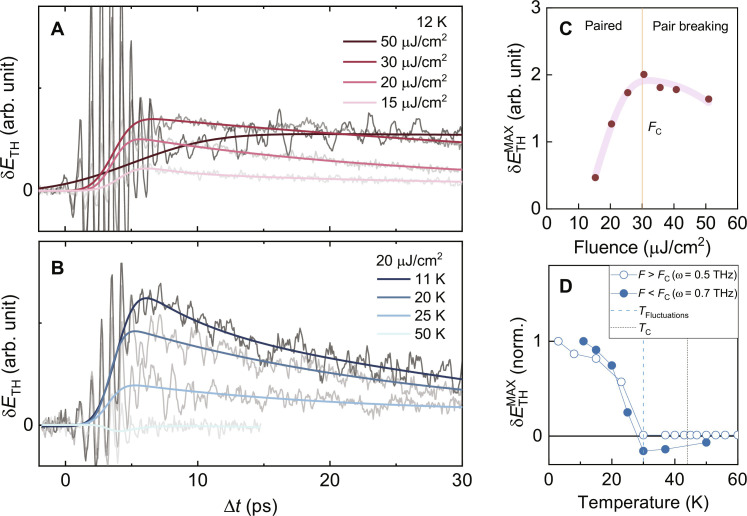
Transient dynamics of the TH peak field amplitude in photoexcited LSCO. (**A**) Pump fluence dependence at 12 K. (**B**) Temperature dependence at a pump fluence *F*_pump_ = 20 μJ/cm^2^. (**C**) Fluence dependence of the extracted peak field changes. The line shows a fit to the data with a saturation effect and a linear decay that sets in at a critical pump fluence *F*_c_ (red vertical line) (see text S5). (**D**) Temperature dependence of the peak field changes at a pump fluences *F > F*_c_ (open circles) and *F < F*_c_ (filled circles). The blue dashed vertical line indicates the onset temperature for scaling fluctuation frequency as found in (*44*). The black dotted vertical line indicates *T*_c_.

Having characterized the full-2D spectrum, let us focus on the fluence and temperature dependence of the differential TH signal. Therefore, the pump fluence and temperature dependence of the TH response are investigated by optically filtering only for the TH dynamics in 1D pump scan (Fig. 1C). Figure 3 (A and B) shows the dynamics of the maximum TH field changes δ*E*_TH_ (sampled as in Fig. 1B) under the photoexcitation as a function of excitation density and temperature, respectively. To measure the excitation density dependence, in Fig. 3A, we fix the temperature at 12 K, and we sweep the optical pump fluence from 15 to 50 μJ/cm^2^. For the temperature dependence reported in Fig. 3B, we set a pump fluence of 20 μJ/cm^2^ that is high enough to saturate the contribution of excited quasiparticle to the linear THz response [*F* ≥ 7 μJ/cm^2^ (*14*)] but lower than the threshold where a flattening of the pump-induced changes of the linear THz response has been measured (*14*).

As shown in Fig. 3A, as a function of increasing pump fluence, the light-induced changes of TH at long time delays, along with the amplitude of the short-time oscillations, monotonically increase (see text S4). However, the peak value of the TH field change ${\delta E}_{\text{TH}}^{\text{MAX}}$ extracted by the fit procedure (explained in text S2) shows a nonmonotonic behavior as a function of the pump fluence (Fig. 3C). According to Eq. 1, the differential TH signal is affected by the photoexcitation via the combined effects on the local THz field *E*_THz_ and on the nonlinear kernel χ. Pump-probe spectroscopies probing the linear THz response to an optical pulse (*14*) always show a monotonic increase of *E*_THz_. On the other hand, as mentioned above, the nonlinear kernel χ ∼ Δ is expected to be suppressed as the order parameter Δ is quenched by the optical pump, regardless of the exact nature of the collective excitations contributing to the nonlinear response. These observations suggest that the nonmonotonous scaling of ${\delta E}_{\text{TH}}^{\text{MAX}}$ follows from these two competing effects: At low fluence, the increase of ${\delta E}_{\text{TH}}^{\text{MAX}}$ can be ascribed to a reduced screening of the local *E*_THz_ field, but its suppression above a critical value *F*_c_ = 30 μJ/cm^2^ indicates the threshold above which the suppression of the nonlinear kernel overcomes the screening effects. Such a nonmonotonous response resembles the observations in photoexcited excitonic insulators (*42*, *43*), where the decrease above a fluence threshold has been attributed to suppression of the exciton condensate.

To gain further insight into the simultaneous effects due to the quasiparticle excitations and the order-parameter suppression on the TH, we turn to the temperature dependence in Fig. 3B. The extracted differential peak value of the TH signal ${\delta E}_{\text{TH}}^{\text{MAX}}$ at low fluence is shown as closed circles in Fig. 3D. A positive differential TH signal persists up to a temperature of *T* ≈ 30 K and changes its sign for higher temperatures. On further increasing temperature, the negative differential TH response remains small even above *T*_c_ before it vanishes.

Previous scaling analysis of equilibrium THz response has shown that in optimally doped La_1.84_Sr_0.16_CuO_4_(LSCO), the scaling fluctuation frequency becomes nonzero already below *T*_c_, at around 30 K (*44*). Such an onset also appears in the temperature-dependent unpumped TH amplitude response, which shows a strong screening peak at 30 K (see text S6) (*25*). This finding suggests that the sign-change of the pump-induced variation of the TH signal ${\delta E}_{\text{TH}}^{\text{MAX}}$ can be ascribed again to a predominant effect of the local-field screening such that a dip occurs at the same temperature where a peak is found in the dissipative linear THz response (*44*). An average heating effect can be ruled out by the temperature dependence of ${\delta E}_{\text{TH}}^{\text{MAX}}$ in the high-fluence regime *F* > *F*_c_, shown as open circles in Fig. 3D. It traces the same temperature dependence of the low-fluence data up to 30 K, but since it is ultimately dominated by SC condensate suppression, it is barely affected by the local-field screening effects above 30 K so that the ${\delta E}_{\text{TH}}^{\text{MAX}}$ signal remains zero above it.

**Fig. 4.**
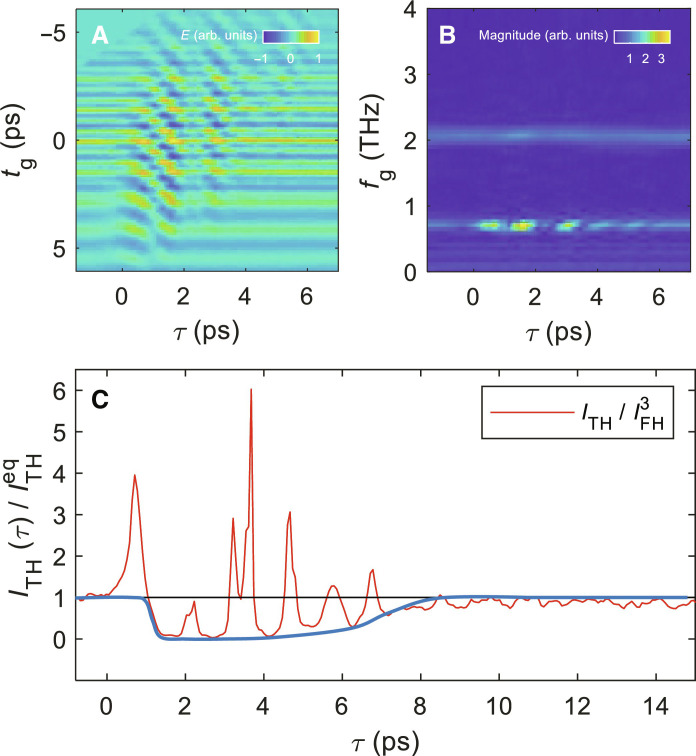
Transient response of the 2D THG signal for the TH field at constant time delay. (**A**) The data of Fig. 2A shearing in *x* axis (∆*t*) so that for each time delay τ = *t*_g_ + ∆*t* the transient THz field has the same delay to the pump pulse. The spectrum is normalized by the maximum value of the transient THz field. (**B**) Fourier transform along the *t*_g_ axis of (A). The spectrum is normalized by the equilibrium value of the FH magnitude. (**C**) Transient dynamics of the normalized TH susceptibility $I_{\text{TH}}/I_{\text{FH}}^{3}$ . The value is again normalized by the dataset of the static case before the pump pulse arrives. The blue solid line is shown as a guide to the eye.

The discussion of the 1D scan shows that to directly access the order-parameter dynamics encoded in the nonlinear kernel, one should be able to get rid of screening effects on the local field and of additional harmonics coming from four-wave mixing with the optical pump. One would expect that slow decay of the δ*E_TH_* response should be ascribed to screening effects, in agreement with direct measurements of pump-induced changes of the linear THz response (*14*), dominated by slow recombination processes of excited quasiparticle. To achieve this goal, we exploit the advantages of a full-2D protocol, focusing on the intrinsic SC order-parameter response under photoexcitation with the low pump fluence of 20 μJ/cm^2^ at 5 K. In the 2D protocol used for Fig. 2A, we keep the distance ∆*t* between the center of the THz pulse and the probe pulse fixed while scanning on the acquisition time *t*_g_. Here, instead, we keep the distance τ between the acquisition time and the pump pulse fixed, as, e.g., done for transient THz spectroscopy (*5*, *7*, *45*) and described, e.g., theoretically in (*46*) (see text S7). Therefore, since τ = *t*_g_ *+* Δ*t* in the 2D measurement of Fig. 2A, we shear the data in the *x* axis (∆*t*), as shown in Fig. 4A, to obtain a 2D scan as a function of *t*_g_ versus τ. Then, we Fourier transform along the gate time *t*_g_ to reveal the transient spectrum in Fig. 4B. We stress that Figs. 2A and 4A are equivalent, and so are their Fourier-transformed spectra, as shown by direct comparison of Fig. 2C and fig. S9D. The main advantage of the (*t*_g_, τ) representation is that here all the additional sideband modulations appear in the FH component as a function of pump-drive delay time τ, while the modulations in the raw TH signal remain weak (see also fig. S9E). The FH modulations show broad frequency contributions around 0.7 to 1.4 THz in a 2D Fourier transform spectrum (see text S7). This broad feature is intrinsically linked to the 45° tilted wave mixing signal in Fig. 2C. The full-2D transient FH and TH responses show the direct observation of the nonlinear current generation by excited quasiparticles with the driving THz field. Now, with the simultaneous determination of FH and TH, we can estimate the pump effects on the nonlinear optical kernel χ by normalizing the TH field amplitude to the transmitted FH field $\chi ∼I_{\text{TH}}/I_{\text{FH}}^{3}$ (*25*, *31*). As shown in Fig. 4C, the envelop of the resulting TH susceptibility $\chi \left(\tau \right)∼I_{\text{TH}}\left(\tau \right)/I_{\text{TH}}^{\text{eq}}$ shows now a marked suppression at short time delays, along with modulations originating from the FH signal in Fig. 4B. Once eliminated, the screening effects on the local electric field, χ(τ), trace the transient SC order-parameter response Δ(τ), with a full suppression of the signal within 1 ps and a relaxation back to the equilibrium value in about 9 ps. These timescales are well in agreement with the fast picosecond suppression and recovery of the SC condensate under photoexcitation probed by TR-ARPES for other cuprate superconductors (*47*). Although we cannot resolve the different contributions of amplitude and phase fluctuations of the SC order parameter made possible by TR-ARPES, we are nonetheless able to show that 2D transient spectra directly probe the fast dynamics of the condensate without spurious effects due to quasiparticle-excitation processes present in 1D scans.

## DISCUSSION

Probing the transient nonlinear THG response of light-driven LSCO provides crucial insight into the multiple contributions of the SC nonlinearities and pair breaking processes to the THG signal. While static THG is a powerful tool to access the thermal evolution toward a coherent SC state (*15*–*24*), we aim here to establish and validate transient THG changes in an optical pump–THz drive scheme as a powerful spectroscopic tool to probe the system under photoexcitations. Thanks in particular to the implementation of 2D protocols, we are able to separate two concomitant effects: the creation of an incoherent, long-lasting quasiparticle population and the emergence of a much faster quench and recover of the order parameter.

Modulations of the signal at 2Ω and 4Ω are fully understood as sideband modulation of the signal due to four-wave mixing among the optical pump and the THz probe field. The excitation density– and temperature-dependent measurements show how the pump-induced quasiparticle excitations modify the screening of the local field, accounting for the THG increase in the low-fluence regime and for its sign-change across the onset temperature for thermal fluctuations, where both the onset temperature and pair-breaking thresholds are in agreement with existing literature. At larger fluence, the THG signal becomes suppressed, signaling the suppression of the SC condensate with a reduction of the THG. In contrast to linear spectroscopy, our probing scheme allows us to identify the pure effects ascribed to suppression of SC order, which dominates the 1D scans above a fluence threshold and is responsible for the nonmonotonic behavior. The effect becomes clear in the 2D scans, where we take advantage of the time evolution of both FH and TH signals to extract the pure nonlinear kernel contribution to THG. The extracted THG dynamics shows a suppression and recovery over a very fast (~9 ps) timescale. As mentioned above, it is well accepted that the nonlinear optical kernel in superconductors grows below *T*_c_ along with the increase of the SC order parameter ∆. As a consequence, the suppression of the THG signal in 2D scans is likely to be due to a direct suppression of the intrinsic order-parameter amplitude (Higgs mode), reflecting a decrease in the overall scale of the nonlinear response. The direct comparison with TR-ARPES data (*47*), where similar suppression and recovering dynamics have been reported, provides a benchmark of the ability of 2D THG spectroscopy to directly access the pump-induced quench of the SC order. On a similar avenue, 2D THz experiments with broadband pulses (*48*, *49*) could serve as probes for the transient regime. 2D transient THG spectroscopy thus offers an additional tool to investigate the quenching and recovery of the Higgs and phase excitations of the SC condensate in unconventional superconductors and sheds light on the fundamental pairing mechanism at play in these systems.

## MATERIALS AND METHODS

### Sample preparation

The optimally doped LSCO sample was grown by molecular beam epitaxy at the Max Planck Institute for Solid State Research. The sample is 80 nm thick on a LaSrAlO_4_ (LSAO) substrate. *T*_c_ was determined at 45 K from mutual inductance measurement.

### Experimental design

We performed an optical pump and THG drive experiment with THz sources based on a femtosecond laser system. For the THG, broadband THz radiation was generated through tilted pulse front scheme using lithium niobate crystal (*50*–*52*). With initial laser pulse energy around 1.5 mJ at 800-nm central wavelength and 100-fs pulse duration, broadband THz radiation with up to 3-μJ pulse energy was generated. To produce narrowband THz radiation, corresponding band-pass filters were used; the setup of THG scheme is detailed in (*25*). In the photoexcitation measurement with a near-infrared (NIR) pump pulse, a 1D pump-TH drive scheme shown in Figs. 1C and 3 was performed by varying the time delay between the pump pulse and the gate pulse by moving the pump pulse. On the other hand, a 2D pump-TH drive experiment was performed by sweeping the THz pulse with a fixed time delay between the THz pulse and the NIR pump pulse with a fixed gate pulse. See text S1 for more information.

## Supplementary Materials

### This PDF file includes:

Supplementary Text Figs. S1 to S9 References
