# Supplementary material for: Tracing the dynamics of superconducting order via transient terahertz third-harmonic generation
Source: Sci Adv. 2024 Mar 15;10(11):eadi7598. doi: 10.1126/sciadv.adi7598 (PMC10942118; doi:10.1126/sciadv.adi7598)
Supplement: Supplementary file 1 — Supplementary Text Figs. S1 to S9 References [file sciadv.adi7598_sm.pdf]

Science Advances
AAAS

Supplementary Materials for

Tracing the dynamics of superconducting order via transient terahertz third-harmonic generation

Min-Jae Kim *et al.*

Corresponding author: Min-Jae Kim, mj.kim@fkf.mpg.de; Lara Benfatto, lara.benfatto@roma1.infn.it; Stefan Kaiser stefan.kaiser@tu-dresden.de

Sci. Adv. **10**, eadi7598 (2024)
DOI: 10.1126/sciadv.adi7598

This PDF file includes:

Supplementary Text
Figs. S1 to S9
References

Supplementary Text S1: Two methods for measuring the TH dynamics under the photoexcitation

The Third harmonic (TH) field driven by high-field multi-cycle THz pulse is a preferential spectroscopic tool to investigate the superconducting order. By applying a combination of an optical pump pulse and a multicycle TH pulse, we investigate non-equilibrium superconductivity. Unlike the conventional time-domain THz probe spectroscopy, which represents the non-equilibrium linear electronic properties, the TH spectroscopy allows us to disentangle the nonlinear properties of the system from the pump-induced effects on the linear response, as due to quasiparticle excitations. We termed our protocol an *optical pump – TH drive* experiment, as opposed to a usual pump-probe scheme.

Tilted pulse front technique generates single-cycle broadband THz field. We filtered it to obtain a narrow-band driving field with the frequency centered at ω=0.7 THz and average duration about 10 ps by using band-pass filter. To measure the driven TH electric field change under the photoexcitation, we focused the optical pump beam with the pulse duration of 100fs onto the sample with a beam diameter 600 µm comparable to the focused THz pulse 560 µm. The data acquisition processes were in two ways, 1) Scanning pump delay (1D scan) and 2) Scanning pump and THz delay lines simultaneously (2D scan).

Figure S1 (a) depicts the 1D scan. Here, t_{THz} and t_{g} are kept fixed to record the change of the TH field while sweeping Δt. For this measurement it is necessary to fully suppress the leaked fundamental driving field (FH) field. We achieve this by installing two bandpass filters (2.1 THz) after the sample. The optical pump beam is modulated by a chopper obtaining the relative changes of the TH field.

Figure S1 (b) shows the 2D scan scheme to collect the full THz spectrum. The static transmitted THz field is scanned by moving the t_{THz} with the fixed gate time delay t_{g}. To collect the full THz spectrum including both FH and TH components, we used the one bandpass filter (2.1THz) after the sample. While the scanning, the optical pump pulse t_{pump} moves with the fixed pump-drive time delay Δt simultaneously. Each scans are operating with systematically varying Δt. In this procedure, we obtained the nonlinear sample response between the THz field and the optical pump pulse.

Supplementary Text S2: Interference effect between optical pump and TH drive field

Figure S2 describes the way of extracting the photoexcited interference feature in a superconductor. At the early pump-drive time delay Δt a prominent strong oscillatory signal emerges on top of the background response, as seen in Fig. S2 (a,b). To extract the oscillatory signal, we fit the non-oscillatory background signal in analogy to previous studies on other correlated materials (53-55). The fit function is designed as

$$\delta E_{TH} = (1 + \mathrm{erf}\left(\frac{\Delta t - \Delta t_0}{\sqrt{2}\tau_{el}}\right))(A_1 \exp\left(-\frac{\Delta t - \Delta t_0}{\tau_1}\right) + A_2 \exp\left(-\frac{t - \Delta t_0}{\tau_2}\right)) + c\,, \quad \text{(S1)}$$

where Δt is the time delay, τ_{el} is the time constant for the excitation of photo-carriers, $\tau_1$and $\tau_1$are the time constant of the exponential decay, and A_1, A_2, c, Δt_0 are constants. Subtracting the fits results in Fig. S2 (c,d). As explained at the end of the previous section, in the 1D pump scan we select only the ω_g= 3Ω dynamics of δE_{TH} by adding an additional band-pass filter. As a consequence the frequency of the Δt oscillations are $\omega_{\Delta t} = \pm 2\Omega$ (1.4 THz) and $\omega_{\Delta t} = \pm 4\Omega$ (2.8 THz), see Fig. S2 (e,f). Apart from the (non-oscillatory) contribution of the static THG signals, Fig. S2 (c,d) are equivalent to the projection of the 2D scans with digitally filtered to obtain only TH component as described in the Supplementary Text S3.

Supplementary Text S3: Theoretical calculation of pump-TH drive dynamics

To pinpoint the origin of THG modulations with even multiples of Ω as reported in the main text, we recorded spectra of the transmitted signal as a function of gate time t_g and pump-drive delay Δt. The resulting spectrum is shown in Fig. S3 (a). Fig. S3 (b-c) show the data after a Fourier transform first along the t_g axis, and then along the Δt-axis, with corresponding frequency variables ω_g and $\omega_{\Delta t}$, respectively. In what follows, we will first focus on the discussion of the experimental data in (ω_g,$\omega_{\Delta t}$)-space shown in Fig. S3 (c). The experimentally recorded transmitted field is directly related to the induced current $j(\omega) = j^{(1)}(\omega) + j^{(3)}(\omega)$ which we further decompose into first- and third-order contributions of the applied electromagnetic vector potential $A = A_{THz} + A_{pump}$. Signatures of the fifth-order current $j^{(5)}(\omega)$ are also observed, as detailed in the below.

The experimental setup consists of three pulses. The optical pump A_{pump}, the THz drive A_{THz}, and a gate pulse at which the parallel electric field component of the transmitted beam is recorded. Here we define the time origin $t = t_{THz} = 0$ to be the center of the THz drive. We denote functional forms of the pulse shapes centered around the origin by a bar, so that $A_{pump}(t) = \bar{A}_{pump}(t + \Delta t)$. In frequency space the pump delay Δt hence results in a phase shift $A_{pump}(\omega) = \bar{A}_{pump}(\omega)e^{-i\omega\Delta t}$. The linear response current is given by

$$j^{(1)}(\omega_g) = T(\omega_g)\chi^{(1)}(\omega_g)A(\omega_g) \approx \chi^{(1)}(\omega_g)A_{THz}(\omega_g)\ , \quad \text{(S2)}$$

where $\chi^{(1)}$ is the linear susceptibility and $T(\omega)$ is the transmission function of a 3Ω-bandpass filter shown in Fig. S4. Only the probe pulse generates a response in the THz regime. Since A_{THz} is unaffected by Δt and is a narrowband multi-cycle pulse centered at frequency Ω, the linear response yields the signal at $(\omega_g, \omega_{\Delta t}) = (\pm\Omega, 0)$ in Fig. S3 (c).

The third order current response is given by the general expression (56-58)

$$j^{(3)}(\omega_g) = T(\omega_g)\int d\omega_1 d\omega_2 \chi^{(3)}(\omega_g, \omega_1, \omega_2)A(\omega_1)A(\omega_2)A(\omega_g - \omega_1 - \omega_2)\ , \quad \text{(S3)}$$

where $\chi^{(3)}$ is the nonlinear susceptibility that captures all microscopic details of the sample, such as the quasiparticles and the Higgs contributions. Decomposing the vector potential as $A = A_{THz} + A_{pump}$, we obtain four different contributions (Fig. S5). The terms $j^{(3)} \sim A_{THz}^2 A_{pump}$ (Fig. S5 (a)) and $j^{(3)} \sim A_{pump}^3$ (Fig. S5 (b)) are odd in orders of the pump pulse and vanish in the THz regime. $j^{(3)} \sim A_{THz}^3$ (Fig. S5 (c)) is the usual pump-independent THG response that gives the $(\omega_g, \omega_{\Delta t}) = (\pm 3\Omega, 0)$ signal in Fig. S3 (c), as well as an additional contribution at $(\omega_g, \omega_{\Delta t}) = (\pm\Omega, 0)$ (56-58). The last term $j^{(3)} \sim A_{THz}A_{pump}^2$ (Fig. S5 (d)) deserves a more detailed discussion. Approximating the narrowband THz probe as $A_{THz}(\omega) = A_{THz}^0(\omega)\delta(\omega \pm \Omega)$, we obtain from Eq. (S3)

$$j^{(3)}(\omega_g, \Delta t) = T(\omega_g)\int d\omega_2 \chi^{(3)}(\omega_g, \pm\Omega, \omega_2)A_{THz}^0(\pm\Omega)\bar{A}_{pump}(\omega_2)\bar{A}_{pump}(\omega_g \mp \Omega - \omega_2)e^{-i(\omega_g \mp \Omega)\Delta t}. \quad \text{(S4)}$$

The Δt-dependence of the nonlinear current is solely manifest in the $e^{-i(\omega_g \mp \Omega)\Delta t}$ phase. The Fourier component of the current at frequency ω_g hence shows phase oscillations in Δt with frequency $\omega_g \pm \Omega$. Once the signal is further Fourier transformed with respect to Δt one readily obtains from Eq. (S4) the continuous signal along the diagonal lines at $\omega_{\Delta t} = \omega_g \pm \Omega$ in Fig. S3 (c). The width

of the signal is limited to the regime $(A_{pump} \otimes A_{pump})(\omega_g \pm \Omega)$ in the ω_g-direction, where $\otimes$ denotes a convolution.

For the optical pump pulse used in the experiment, $A_{pump} \otimes A_{pump}$ has an approximately Gaussian peak centered around zero with FWHM of ~5 THz, consistent with the broad diagonal feature observed between $|\omega_g| \lessapprox 3\Omega$. The absence of signal at low frequencies (between $|\omega_g| \lessapprox \Omega$) in Fig. S3 (f) is a consequence of the 3Ω bandpass filter used in the experiment. We can intuitively understand Eq. (S4) within the diagrammatic representation shown in Fig. S5. Here, one photon of the THz drive pulse (black wavy line) combines with two photons of the pump pulse (blue wavy lines) to produce the nonlinear current (red wavy line). The full grey bubble represents the nonlinear susceptibility $\chi^{(3)}$ that embodies the microscopic details of the material. In the case of both the quasiparticle and the Higgs mode contributions, such renormalization would lead to a peak in the susceptibility at the characteristic resonance energy of 2Δ. To conserve energy, the frequency of the three photons must match the frequency of the induced current. The probe photon can only contribute with energies $\pm\Omega$. While the photon energies of the pump pulse lie in the optical regime, i.e. $\nu \sim \pm$eV, their frequency spectrum is rather broad and two such high energy photons can combine in a difference frequency (DF) process to yield the total energy $\omega \mp \Omega$ in the THz regime. Due to the pump delay Δt, both pump photons pick up a phase that results in phase oscillations in Δt manifest in the diagonal spectral features at $\omega_{\Delta t} = \omega_g \pm \Omega$ in Fig. S3 (c) (marked by the green rectangles boxes in Fig. 2 (c) in the main text). Following the same steps detailed above, the diagonal stripes at $\omega_{\Delta t} = \omega_g \pm 3\Omega$ in Fig. S3 (c) (marked by the red rectangle box in Fig. 2 (c) in the main text) can be related instead to the fifth-order current $j^{(5)}$.

The 2D experiment described above differs from the 1D experiment described in the main text in two ways. First, the 1D experiment was performed at two selected values of the gate time $t_g = t^{\pm}$, corresponding to the maximum(minimum) field strength of the THz probe pulse. Second, the 2D experiment was performed using a single 3Ω bandpass filter of transmission $T(\omega)$, see Eq. (S2), while two such filters, $T(\omega)^2$, were used in the 1D experiment. The experimentally recorded transmission function of the filter is shown in Fig. S4. To verify the consistency of the two experiments, we bridge these difference in two steps. We first digitally apply the second filter to the 2D data according to

$$j^{(3)}_{filtered}(\omega_g) = T(\omega_g) j^{(3)}(\omega_g) \quad \text{(S5)}$$

The filtered data in the $(\omega_g, \omega_{\Delta t})$-domain are shown in Fig. S3 (f). We see that the remaining signal is concentrated in a narrow band around $\omega_g = \pm 3\Omega$. Fourier transforming the axes back into time domain yields Fig. S3 (d-e).

Next, we tune the gate time t_g to the maximum (minimum) $t^{\pm}$ of the signal as indicated by blue (orange) lines in Fig. S3 (d). In principle $t^{\pm}$ should be independent of Δt, but we allow for a small Δt-dependence to compensate for small drifts of the pulse position when Δt is experimentally scanned.

A plot of the signal along the blue (orange) lines is shown in Fig. S6 (a). We observe oscillations in Δt in agreement to the 1D data of Fig. S2 (a,b) (Fig. 3 (a,b) in the main text). The corresponding Fourier transform in Fig. S6 (b) reveals the frequency of these oscillations to be 2Ω and 4Ω.

From the preceding discussion, we can understand the origin of the 2Ω and 4Ω oscillations. The measurement for a specific gate time $t^{\pm}$ corresponds to an integration over a range of ω_g frequencies in Fourier space. This is approximately equivalent to a projection of the spectrum in

Fig. S3 (c) on the $\omega_{\Delta t}$-axis. However, the presence of the filter removes all but the $\omega_g = \pm 3\Omega$ signal. Hence, mostly the signal at $\omega_g = \pm 3\Omega$ contributes. Therefore, the 1D measurement effectively probes the points $(\omega_g, \omega_{\Delta t}) = (\pm 3\Omega, 0)$, $(\pm 3\Omega, \pm 2\Omega)$, and $(\pm 3\Omega, \pm 4\Omega)$ in the 2D FT spectrum shown in Fig. S3 (f). The same effect is easily seen in time domain in the 1D differential scans reported in Fig. 1 (c) and Fig. 3 (a,b) of the main text. In this case the THz field-change δE_{TH}scales as the amplitude of the pump-induced contribution $j^{(3)}(\omega_g, \Delta t)$ to the non-linear current given by Eq. (S4). It is then expected that it oscillates as a function of Δt at 2Ω and 4Ω, the latter being suppressed by the filtering procedure as shown by comparing Fig. S3 (c) and (f).

Supplementary Text S4: The light-induced changes of TH at long time delays

Figure S7 shows the light-induced changes in TH at a pump-probe delay of Δt = 60 ps. According to Eq. (1), one would expect the signal to scale with E^2_{pump}.i.e. with the pump fluence. Such a behavior is indeed observed for the signal at long time delays, where the TH amplitude changes show a linear dependence on optical pump flunence F ($\propto E^2_{pump}$) as it increases. Additionally, the absence of saturation behavior suggests that we may not have reached the saturation regime, as clearly demonstrated at pump fluences exceeding 50 μJ/cm^2 [14].

Supplementary Text S5: The non-monotonic behavior of the maximum peak-field changes of TH as a function of the pump fluence

The maximum peak-field changes of the TH signal, δE^{MAX}_{TH} of Fig. 3C, exhibit a non-monotonic behavior as a function of fluence F. As the pump fluence increases, a non-linear increase feature is evident, resembling observations in photoexcited optical and linear THz responses, suggesting a saturation behavior indicative of the destruction of superconductivity [14]. Also, a comparable depletion feature above a critical pump fluence has been found in photo-excited excitonic insulators [42, 43]. In analogy to these previous studies, the fit function is designed as

$$\delta E^{MAX}_{TH} = C_1 * \left(1 - \mathrm{erf}\left(\frac{-F}{F_c}\right)\right) - C_2 * (1 + \mathrm{erf}\left(\frac{F-F_c}{w}\right)) * (F - F_c) + d. \quad \text{(S6)}$$

The first term corresponds to the saturating part, where F denotes the optical pump fluence, and F_c characterizes the position of the critical fluence. The second term in Eq. (S6) describes the linear decrease behavior, where w representing the width. The factors C_1, C_2 describe the amplitudes of the two components, and d is a constant.

Supplementary Text S6: Comparison the pump induced TH changes to unpumped TH amplitude

Figure S8 shows the direct comparison of the TH field changes under the photoexcitation to the unpumped TH field response. The onset of the TH field changes signal below 30K appears at temperatures where the unpumped TH field intensity peaks. As discussed in ref. 25 this suppression of the static TH signal is due to strong screening effects that take place in the superconducting phase. This finding suggests that the sign-change of the pump- induced variation of the TH signal can be explained by a predominant effect of the local-field screening.

Supplementary Text S7: 2D pump-drive spectra along sheared time axis τ

To examine the features of the transient dynamics of the linear FH component and the nonlinear TH signal distinctly, we now consider a different scheme: namely, we introduce a new variable $\tau = t_g + \Delta t$ and plot the 2D spectra as a function of (t_g, τ) instead of $(t_g, \Delta t)$, as shown in Fig. S9 (b). Geometrically, this corresponds to a 45° shear of the original 2D spectrum in Fig. 2A. This physically corresponds to collecting individually traces of the experimental data at fixed pump-gate delay, or adopting the method 2 described in Ref. 46. This is equivalent to the experimental scheme shown in Fig. S9 (a). By fixing the time between the pump pulse and gate pulse and sweeping both

pulses while the THz pulse is fixed, every point of the THz waveform is measured with same pump-probe delay time τ.

As shown in Fig. S9 (c) after the Fourier transform along the gate time t_g, the FH now shows coherent modulations as a function of pump-drive delay time τ (Plots of the FH and TH intensity changes are shown in Fig. S9 (e)). The spectral feature of background excitation-relaxation dynamics are marked by cigar-shaped contours (blue: FH, violet: TH). These features are tilted by 45° compared to the corresponding in the $(f_g, f_{\Delta t})$-representation of (Fig. 2C). In a similar vein, the horizontal feature at the FH component shown in frequency with broadened from ω to 2ω marked by green rectangle in Fig. S9 (d). In the tiled 2D spectra shows c The FH modulations indicates that the FH modulations is highly related on the TH modulations.

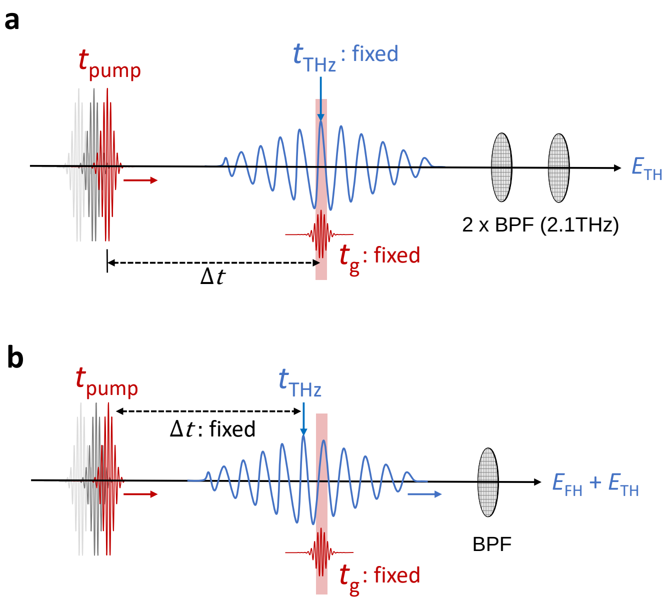

Fig. S1. Experimental scheme for the optical pump-TH drive experiment. The three time points t_{pump}, t_{THz} and t_g are shown. t=t_{THz}=0 defines the center of the THz envelope and it is set conventionally to zero. The center t=-t_{pump} <0 of the pump pulse is located at negative times, and we denote the pump probe delays as Δt= t_{THz} - t_{pump}. In this convention, Δt is always positive when the pump pulse precedes the THz probe. (a) 1D pump scheme. t_{THz} and t_{gate} are fixed to record the change of the TH field while sweeping Δt. To suppress the dominant FH field, two band pass filter (BPF) are installed after the sample. Here, Δt changes in one scan. (b) 2D time-domain scans scheme. The t_{pump} and t_{THz} are simultaneously varying while the t_g =0 is fixed. The Δt is fixed during a scan in t_{gate}, subsequently each scan has a different value for the time distance Δt.

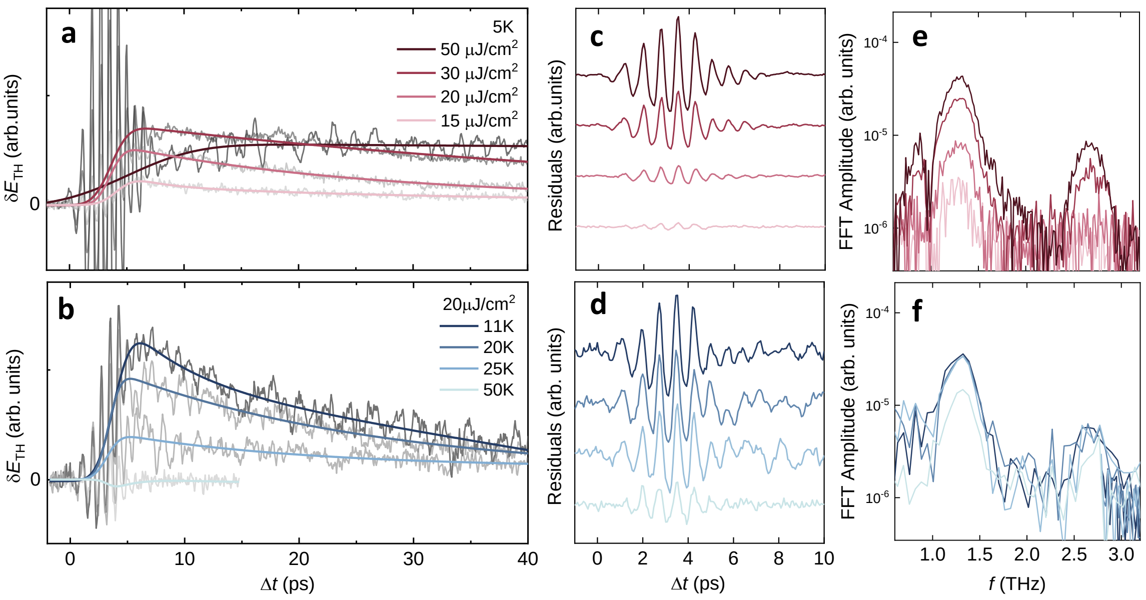

Fig. S2. Transient dynamics of the TH peak field under the photoexcitation. (a,b) 1D pump-probe spectra of the experimentally measured photoexcited superconductivity probing nonlinearly driven TH signal in solid lines with greyscale. The fit curves are plotted with colored solid lines. (c,d) The coherent modulations are extracted by the fit of pairing amplitude dynamics. The FFT amplitude of the modulations are represented in (e) and (f).

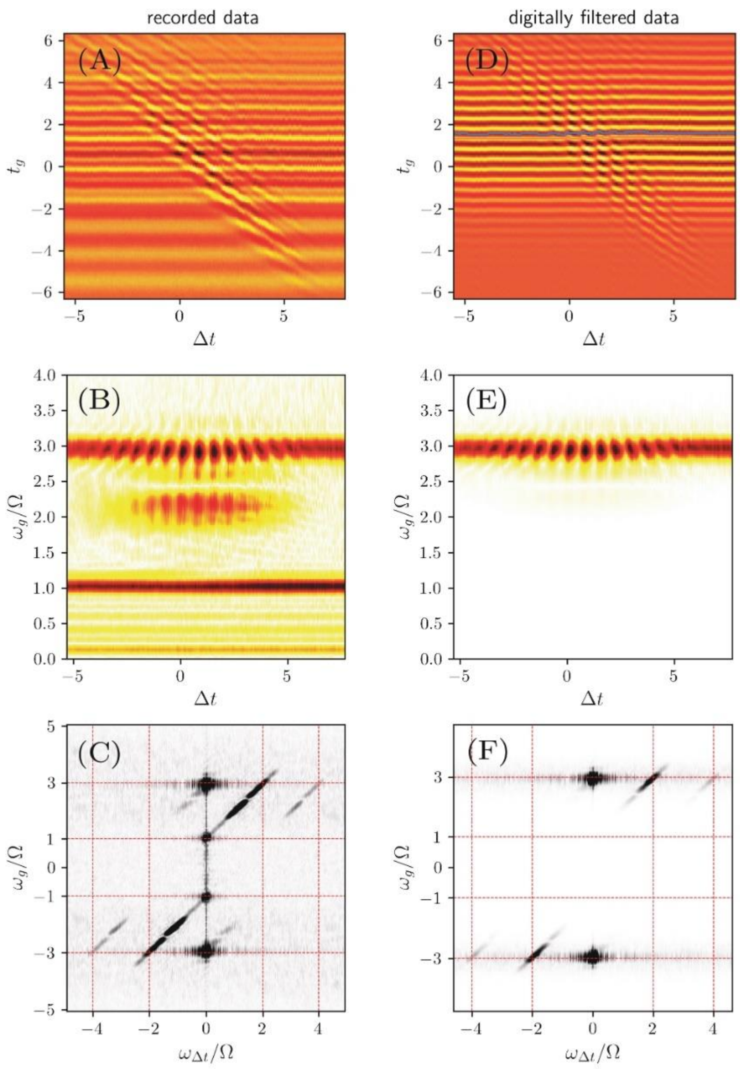

Fig. S3. 2D spectra in time and frequency domain. (a-c) Experimentally recorded data for 2D scans in (a) time domain, (b) time-frequency domain and (c) frequency domain. The experiment was performed using a single 2.1 THz band pass filter, so that leakage of FH can be observed. (d-f) Projection of the 2D scan results on the experimental conditions used to acquire the 1D scans. Since in 1D scans two band-pass 2.1 THz filters have been used, a second 2.1 THz band pass filter has been digitally applied to the data of panel (a-c) to match the experimental setup used to produce the data shown in Fig. 1(c) of the main manuscript and in Supplementary Text S2. This completely filters out the FH signal, as seen in panels (e) and (f). In addition, the second filter also removes the TH modulations at $\omega_{\Delta t} = 4\Omega$ that are still visible in panel (f). The data Fourier transformed back to real time are shown in panels (d) and (e).

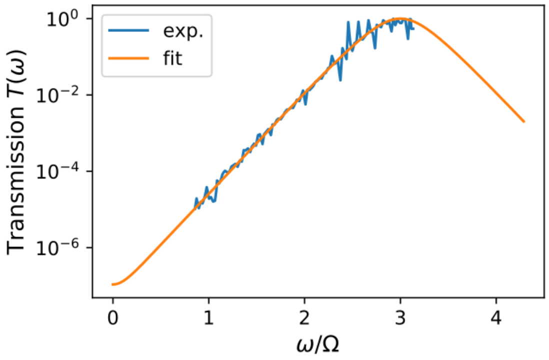

Fig. S4. Transmission function $T(\omega)$ of the band pass filter. Blue curve shows the experimentally recorded data, orange curve shows a fit used in the data processing routine.

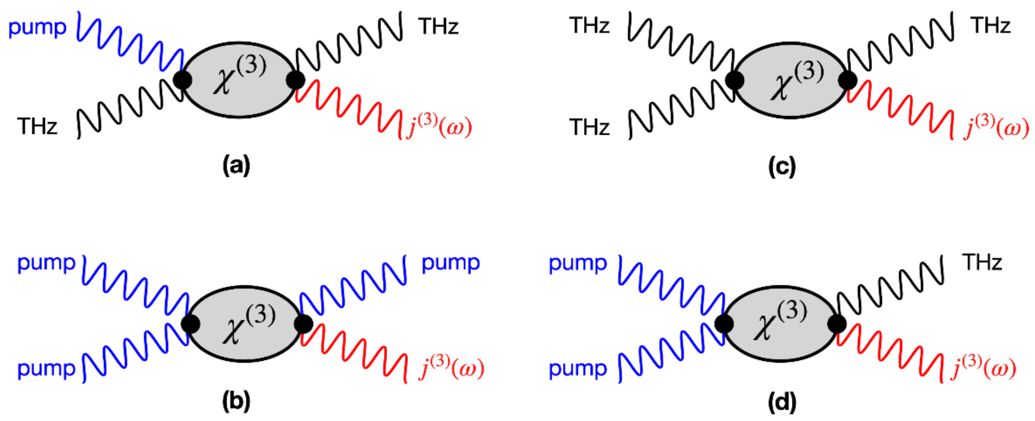

Fig. S5. Diagrammatic representation of the non-linear response. Diagrammatic representation of all the contributions to the third-order nonlinear current $j^{(3)}(\omega)$ (red wiggly line). The third-order susceptibility $\chi^{(3)}$, including in principle all diamagnetic, paramagnetic and mixed terms in the presence of disorder (see Ref. 56-58 for more details), is schematically represented here as a full grey bubble. (a) Process involving two THz and one optical pump photon: the nonlinear current response in this case is in the optical range as the pump field. (b) FH and TH generation process of the optical pump field. (c) FH and TH processes of the THz field, which provides the contributions to the Ω and 3Ω signals measured in the experiments. (d) Process involving two pump photons (blue) and one THz photon (black): in order to obtain a THz response a difference-frequency process involving the two pump photons needs to be considered.

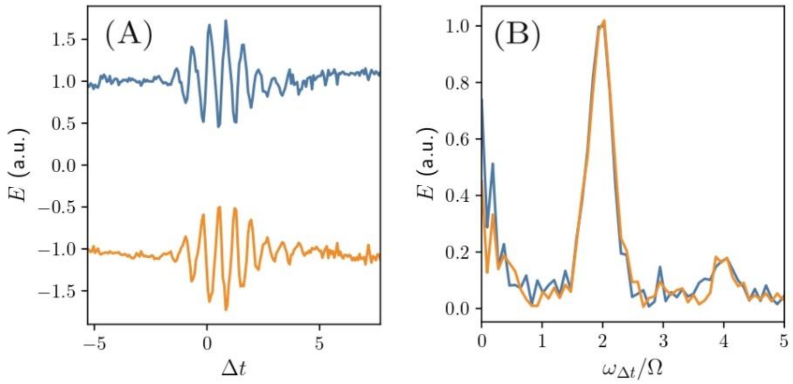

Fig. S6. Analyzing dynamic patterns of the digitally filtered data in Fig. S3 (d). (a) Plot of the maximum (blue) and minimum (orange) of the digitally filtered data of Fig. S3 (d) as a function of Δt. The data correspond to the slice marked by horizontal lines in Fig. S3 (d). The Fourier transform of the data with respect to Δt in (b) reveals 2Ω and 4Ω components of the modulations in Δt.

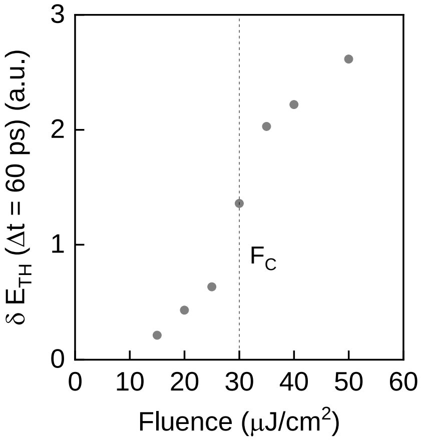

Fig. S7. Fluence dependence of the TH peak field changes at long time delay. Optical pump fluence dependence of the extracted TH peak-field changes at the pump-probe delay time Δt=60ps.

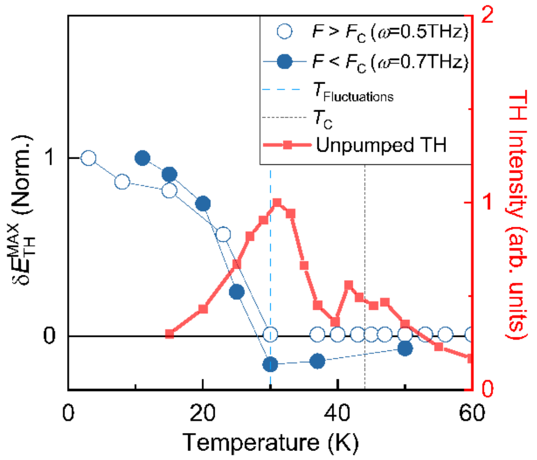

Fig. S8. Comparison between the unpumped and pumped TH signal. Temperature dependence of the TH peak field changes and unpumped TH field intensity. The blue data (left axis) shows the TH peak field changes under the photoexcitation as discussed in the main text (Fig. 4 (d)) and the red data (right axis) shows the TH intensity without optical pump from Ref. 25.

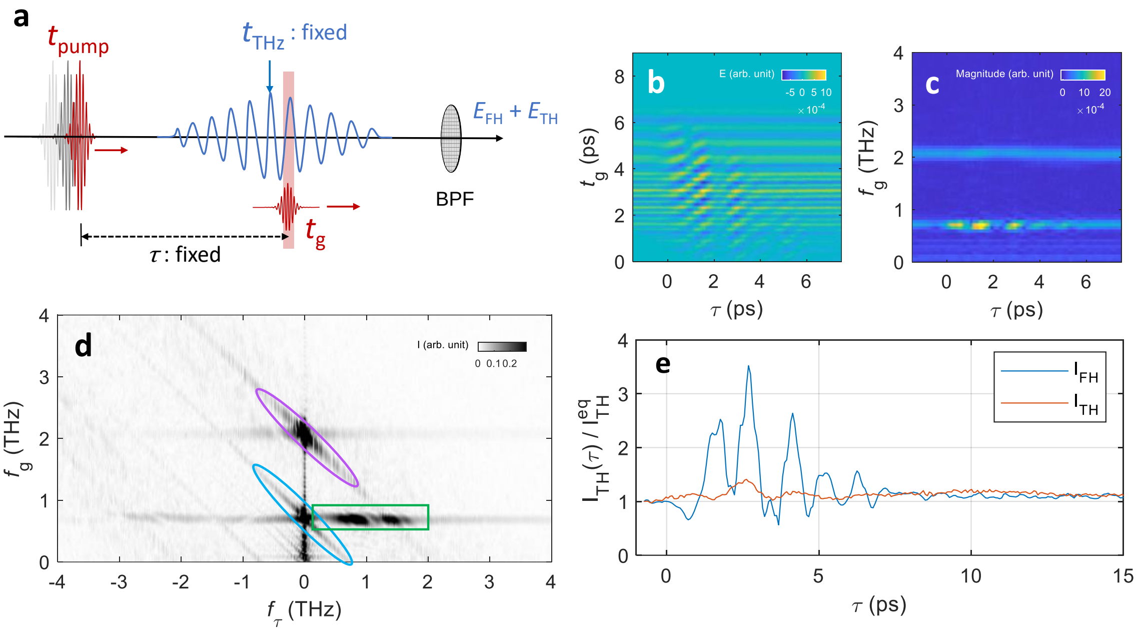

Fig. S9. Intrinsic response of optical pump-TH drive signal. (a) Equivalent experimental scheme to shearing 2D spectrum. The t_{pump} and t_{gate} are simultaneously varying while the t_{THz} =0 is fixed. The time difference between the pump and the gate, τ, is fixed during a scan, subsequently each scan has a different value for the time distance $\tau = t_g + \Delta t$. (b) The sheared data in x-axis (Δt) of Figure 2(a). The spectrum is normalized by the maximum value of the transient THz field. (c) Fourier transform along the t_g axis of (a). The spectrum is normalized by the equilibrium value of the FH magnitude. (d) A 2D Fourier transform of (b) along the sampling t_g and pump-drive delay axis τ. Cigar- and rectangular- shaped features correspond to pumped dynamics and emerged photoexcited harmonics, respectively. (e) Transient dynamics of the FH and TH intensity (I_{FH}, I_{TH}) extracted from (c). The value is normalized by the dataset of the static case before the pump pulse arrives.

REFERENCES

1. A. Kirilyuk, A. V. Kimel, T. Rasing, Ultrafast optical manipulation of magnetic order. *Rev. Mod. Phys.* **82**, 2731–2784 (2010).

2. C. Jia, N. Zhang, A. Sukhov, J. Berakdar, Ultrafast transient dynamics in composite multiferroics. *New J. Phys.* **18**, 023002 (2016).

3. M. S. El Hadri, M. Hehn, G. Malinowski, S. Mangin, Materials and devices for all-optical helicity-dependent switching. *J. Phys. D Appl. Phys.* **50**, 133002 (2017).

4. A. V. Kimel, A. M. Kalashnikova, A. Pogrebna, A. K. Zvezdin, Fundamentals and perspectives of ultrafast photoferroic recording. *Phys. Rep.* **852**, 1–46 (2020).

5. D. Fausti, R. I. Tobey, N. Dean, S. Kaiser, A. Dienst, M. C. Hoffmann, S. Pyon, T. Takayama, H. Takagi, A. Cavalleri, Light-induced superconductivity in a stripe-ordered cuprate. *Science* **331**, 189–191 (2011).

6. S. Kaiser, Light-induced superconductivity in high-T_c cuprates. *Phys. Scr.* **92**, 103001 (2017).

7. S. Kaiser, C. R. Hunt, D. Nicoletti, W. Hu, I. Gierz, H. Y. Liu, M. Le Tacon, T. Loew, D. Haug, B. Keimer, A. Cavalleri, Optically induced coherent transport far above T_c in underdoped $YBa_2Cu_3O_{6+\delta}$. *Phys. Rev. B* **89**, 184516 (2014).

8. B. Liu, M. Först, M. Fechner, D. Nicoletti, J. Porras, T. Loew, B. Keimer, A. Cavalleri, Pump frequency resonances for light-induced incipient superconductivity in $YBa_2Cu_3O_{6.5}$. *Phys. Rev. X* **10**, 011053 (2020).

9. B. Keimer, S. A. Kivelson, M. R. Norman, S. Uchida, J. Zaanen, From quantum matter to high-temperature superconductivity in copper oxides. *Nature* **518**, 179–186 (2015).

10. R. A. Kaindl, M. Woerner, T. Elsaesser, D. C. Smith, J. F. Ryan, G. A. Farnan, M. P. McCurry, D. G. Walmsley, Ultrafast mid-infrared response of $YBa_2Cu_3O_{7-\delta}$. *Science* **287**, 470–473 (2000).

11. L. Stojchevska, P. Kusar, T. Mertelj, V. V. Kabanov, Y. Toda, X. Yao, D. Mihailovic, Mechanisms of nonthermal destruction of the superconducting state and melting of the charge-density-wave state by femtosecond laser pulses. *Phys. Rev. B* **84**, 180507 (2011).

12. A. Pashkin, M. Porer, M. Beyer, K. W. Kim, A. Dubroka, C. Bernhard, X. Yao, Y. Dagan, R. Hackl,

A. Erb, J. Demsar, R. Huber, A. Leitenstorfer, Femtosecond response of quasiparticles and phonons in superconducting $YBa_2Cu_3O_{7-\delta}$ studied by wideband terahertz spectroscopy. *Phys. Rev. Lett.* **105**, 067001 (2010).

13. M. Beck, M. Klammer, S. Lang, P. Leiderer, V. V. Kabanov, G. N. Gol'tsman, J. Demsar, Energygap dynamics of superconducting NbN thin films studied by time-resolved terahertz spectroscopy. *Phys. Rev. Lett.* **107**, 177007 (2011).

14. M. Beyer, D. Städter, M. Beck, H. Schäfer, V. V. Kabanov, G. Logvenov, I. Bozovic, G. Koren, J. Demsar, Photoinduced melting of superconductivity in the high-Tc superconductor $La_{2-x}Sr_xCuO_4$ probed by time-resolved optical and terahertz techniques. *Phys. Rev. B* **83**, 214515 (2011).

15. T. Cea, C. Castellani, L. Benfatto, Nonlinear optical effects and third-harmonic generation in superconductors: Cooper pairs versus Higgs mode contribution. *Phys. Rev. B* **93**, 180507 (2016).

16. R. Matsunaga, N. Tsuji, H. Fujita, A. Sugioka, K. Makise, Y. Uzawa, H. Terai, Z. Wang, H. Aoki, R. Shimano, Light-induced collective pseudospin precession resonating with Higgs mode in a superconductor. *Science* **345**, 1145–1149 (2014).

17. R. Matsunaga, N. Tsuji, K. Makise, H. Terai, H. Aoki, R. Shimano, Polarization-resolved terahertz third-harmonic generation in a single-crystal superconductor NbN: Dominance of the Higgs mode beyond the BCS approximation. *Phys. Rev. B* **96**, 020505 (2017).

18. Y. Murotani, R. Shimano, Nonlinear optical response of collective modes in multiband superconductors assisted by nonmagnetic impurities. *Phys. Rev. B* **99**, 224510 (2019).

19. G. Seibold, M. Udina, C. Castellani, L. Benfatto, Third harmonic generation from collective modes in disordered superconductors. *Phys. Rev. B* **103**, 014512 (2021).

20. M. Silaev, Nonlinear electromagnetic response and Higgs-mode excitation in BCS superconductors with impurities. *Phys. Rev. B* **99**, 224511 (2019).

21. N. Tsuji, H. Aoki, Theory of Anderson pseudospin resonance with Higgs mode in superconductors. *Phys. Rev. B* **92**, 064508 (2015).

22. N. Tsuji, Y. Murakami, H. Aoki, Nonlinear light-Higgs coupling in superconductors beyond BCS: Effects of the retarded phonon-mediated interaction. *Phys. Rev. B* **94**, 224519 (2016).

23. N. Tsuji, Y. Nomura, Higgs-mode resonance in third harmonic generation in NbN superconductors: Multiband electron-phonon coupling, impurity scattering, and polarization-angle dependence. *Phys.*

Rev. Res. **2**, 043029 (2020).

24. M. Udina, J. Fiore, T. Cea, C. Castellani, G. Seibold, L. Benfatto, THz non-linear optical response in cuprates: Predominance of the BCS response over the Higgs mode. *Faraday Discuss.* **237**, 168–185 (2022).

25. H. Chu, M. J. Kim, K. Katsumi, S. Kovalev, R. D. Dawson, L. Schwarz, N. Yoshikawa, G. Kim, D. Putzky, Z. Z. Li, H. Raffy, S. Germanskiy, J. C. Deinert, N. Awari, I. Ilyakov, B. Green, M. Chen, M. Bawatna, G. Cristiani, G. Logvenov, Y. Gallais, A. V. Boris, B. Keimer, A. P. Schnyder, D. Manske, M. Gensch, Z. Wang, R. Shimano, S. Kaiser, Phase-resolved Higgs response in superconducting cuprates. *Nat. Commun.* **11**, 1793 (2020).

26. T. Yoshida, M. Hashimoto, S. Ideta, A. Fujimori, K. Tanaka, N. Mannella, Z. Hussain, Z. X. Shen, M. Kubota, K. Ono, S. Komiya, Y. Ando, H. Eisaki, S. Uchida, Universal versus material-dependent two-gap behaviors of the high-T_c cuprate superconductors: Angle-resolved photoemission study of $La_{2-x}Sr_xCuO_4$. *Phys. Rev. Lett.* **103**, 037004 (2009).

27. R. Matsunaga, Y. I. Hamada, K. Makise, Y. Uzawa, H. Terai, Z. Wang, R. Shimano, Higgs amplitude mode in the BCS superconductors Nb1−xTixN-induced by terahertz pulse excitation. *Phys. Rev. Lett.* **111**, 057002 (2013).

28. R. Matsunaga, R. Shimano, Nonequilibrium BCS state dynamics induced by intense terahertz pulses in a superconducting NbN film. *Phys. Rev. Lett.* **109**, 187002 (2012).

29. F. Giorgianni, T. Cea, C. Vicario, C. P. Hauri, W. K. Withanage, X. Xi, L. Benfatto, Leggett mode controlled by light pulses. *Nat. Phys.* **15**, 341–346 (2019).

30. X. Yang, C. Vaswani, C. Sundahl, M. Mootz, L. Luo, J. H. Kang, I. E. Perakis, C. B. Eom, J. Wang, Lightwave-driven gapless superconductivity and forbidden quantum beats by terahertz symmetry breaking. *Nat. Photonics* **13**, 707–713 (2019).

31. K. Isoyama, N. Yoshikawa, K. Katsumi, J. Wong, N. Shikama, Y. Sakishita, F. Nabeshima, A. Maeda, R. Shimano, Light-induced enhancement of superconductivity in iron-based superconductor $FeSe_{0.5}Te_{0.5}$. *Commun. Phys. Ther.* **4**, 160 (2021).

32. J. Fiore, M. Udina, M. Marciani, G. Seibold, L. Benfatto, Contribution of collective excitations to third harmonic generation in two-band superconductors: The case of MgB_2. *Phys. Rev. B* **106**, 094515 (2022).

33. T. Cea, P. Barone, C. Castellani, L. Benfatto, Polarization dependence of the third-harmonic

generation in multiband superconductors. *Phys. Rev. B* **97**, 094516 (2018).

34. F. Gabriele, M. Udina, L. Benfatto, Non-linear Terahertz driving of plasma waves in layered cuprates. *Nat. Commun.* **12**, 752 (2021).

35. S. Parham, H. Li, T. J. Nummy, J. A. Waugh, X. Q. Zhou, J. Griffith, J. Schneeloch, R. D. Zhong, G. D. Gu, D. S. Dessau, Ultrafast gap dynamics and electronic interactions in a photoexcited cuprate superconductor. *Phys. Rev. X* **7**, 041013 (2017).

36. K. Katsumi, M. Nishida, S. Kaiser, S. Miyasaka, S. Tajima, R. Shimano, Near-infrared light-induced superconducting-like state in underdoped $YBa_2Cu_3O_y$ studied byc-axis terahertz third-harmonic generation. *Phys. Rev. B* **107**, 214506 (2023).

37. S. Nakamura, K. Katsumi, H. Terai, R. Shimano, Nonreciprocal terahertz second-harmonic generation in superconducting NbN under supercurrent injection. *Phys. Rev. Lett.* **125**, 097004 (2020).

38. C. Vaswani, M. Mootz, C. Sundahl, D. H. Mudiyanselage, J. H. Kang, X. Yang, D. Cheng, C. Huang, R. H. J. Kim, Z. Liu, L. Luo, I. E. Perakis, C. B. Eom, J. Wang, Terahertz second-harmonic generation from lightwave acceleration of symmetry-breaking nonlinear supercurrents. *Phys. Rev. Lett.* **124**, 207003 (2020).

39. R. Grasset, K. Katsumi, P. Massat, H.-H. Wen, X.-H. Chen, Y. Gallais, R. Shimano, Terahertz pulse-driven collective mode in the nematic superconducting state of $Ba_{1-x}K_xFe_2As_2$. *npj Quantum Mater.* **7**, 4 (2022).

40. K. Katsumi, Z. Z. Li, H. Raffy, Y. Gallais, R. Shimano, Superconducting fluctuations probed by the Higgs mode in $Bi_2Sr_2CaCu_2O_{8+x}$ thin films. *Phys. Rev. B* **102**, 054510 (2020).

41. K. Katsumi, N. Tsuji, Y. I. Hamada, R. Matsunaga, J. Schneeloch, R. D. Zhong, G. D. Gu, H. Aoki, Y. Gallais, R. Shimano, Higgs mode in the *d*-wave superconductor $Bi_2Sr_2CaCu_2O_{8+x}$ driven by an intense terahertz pulse. *Phys. Rev. Lett.* **120**, 117001 (2018).

42. D. Werdehausen, T. Takayama, G. Albrecht, Y. Lu, H. Takagi, S. Kaiser, Photo-excited dynamics in the excitonic insulator Ta_2NiSe_5. *J. Phys. Condens. Matter* **30**, 305602 (2018).

43. D. Werdehausen, T. Takayama, M. Höppner, G. Albrecht, W. Rost Andreas, Y. Lu, D. Manske, H. Takagi, S. Kaiser, Coherent order parameter oscillations in the ground state of the excitonic insulator Ta_2NiSe_5. *Sci. Adv.* **4**, eaap8652 (2018).

44. L. S. Bilbro, R. V. Aguilar, G. Logvenov, O. Pelleg, I. Bozovic, N. P. Armitage, Temporal correlations of superconductivity above the transition temperature in $La_{2-x}Sr_xCuO_4$ probed by terahertz spectroscopy. *Nat. Phys.* **7**, 298–302 (2011).

45. R. D. Averitt, G. Rodriguez, A. I. Lobad, J. L. W. Siders, S. A. Trugman, A. J. Taylor, Nonequilibrium superconductivity and quasiparticle dynamics in $YBa_2Cu_3O_{7-\delta}$. *Phys. Rev. B* **63**, 140502 (2001).

46. J. P. Revelle, A. Kumar, A. F. Kemper, Theory of time-resolved optical conductivity of superconductors: Comparing two methods for its evaluation. *Condens. Matter* **4**, 79 (2019).

47. F. Boschini, E. H. da Silva Neto, E. Razzoli, M. Zonno, S. Peli, R. P. Day, M. Michiardi, M. Schneider, B. Zwartsenberg, P. Nigge, R. D. Zhong, J. Schneeloch, G. D. Gu, S. Zhdanovich, A. K. Mills, G. Levy, D. J. Jones, C. Giannetti, A. Damascelli, Collapse of superconductivity in cuprates via ultrafast quenching of phase coherence. *Nat. Mater.* **17**, 416–420 (2018).

48. L. Luo, M. Mootz, J. H. Kang, C. Huang, K. Eom, J. W. Lee, C. Vaswani, Y. G. Collantes, E. E. Hellstrom, I. E. Perakis, C. B. Eom, J. Wang, Quantum coherence tomography of light-controlled superconductivity. *Nat. Phys.*, **19**, 201–209 (2022).

49. Kota Katsumi, Jacopo Fiore, Mattia Udina, Ralph Romero III, David Barbalas, John Jesudasan, Pratap Raychaudhuri, Goetz Seibold, Lara Benfatto, N. P. Armitage, Revealing novel aspects of light-matter coupling in terahertz two-dimensional coherent spectroscopy: The case of the amplitude mode in superconductors. arXiv:2311.16449 [cond-mat.supr-con] (2023).

50. H. Hirori, A. Doi, F. Blanchard, K. Tanaka, Single-cycle terahertz pulses with amplitudes exceeding 1 MV/cm generated by optical rectification in $LiNbO_3$. *Appl. Phys. Lett.* **98**, 091106 (2011).

51. K.-L. Yeh, M. C. Hoffmann, J. Hebling, K. A. Nelson, Generation of 10μJ ultrashort terahertz pulses by optical rectification. *Appl. Phys. Lett.* **90**, 171121 (2007).

52. T. Kampfrath, K. Tanaka, K. A. Nelson, Resonant and nonresonant control over matter and light by intense terahertz transients. *Nat. Photonics* **7**, 680–690 (2013).

53. J. Demsar, J. L. Sarrao, A. J. Taylor, Dynamics of photoexcited quasiparticles in heavy electron compounds. *J. Phys. Condens. Matter* **18**, R281– R314 (2006).

54. G. P. Segre, N. Gedik, J. Orenstein, D. A. Bonn, R. Liang, W. N. Hardy, Photoinduced changes of reflectivity in single crystals of $YBa_2Cu_3O_{6.5}$ (Ortho II). *Phys. Rev. Lett.* **88**, 137001 (2002).

55. N. Gedik, P. Blake, R. C. Spitzer, J. Orenstein, R. Liang, D. A. Bonn, W. N. Hardy, Single quasiparticle stability and quasiparticle-pair decay in $YBa_2Cu_3O_{6.5}$. *Phys. Rev. B* **70**, 014504 (2004).

56. M. Udina, T. Cea, L. Benfatto, Theory of coherent-oscillations generation in terahertz pump-probe spectroscopy: From phonons to electronic collective modes. *Phys. Rev. B* **100**, 165131 (2019).

57. M. Puviani, Quench–drive spectroscopy of cuprates. *Faraday Discussion* **237**, 125–147 (2022).

58. M. Puviani, R. Haenel, D. Manske, Quench-drive spectroscopy and high-harmonic generation in BCS superconductors. *Phys. Rev. B* **107**, 094501 (2023).
